# Serum ghrelin, but not obestatin, is a potential predictor of acute pancreatitis severity

**DOI:** 10.1097/MD.0000000000007963

**Published:** 2017-09-01

**Authors:** Huilin Wang, Mengbin Qin, Zhihai Liang, Renjie Chang, Hongzong Fu, Yule Wei, Guodu Tang

**Affiliations:** a Department of Gastroenterology, The First Affiliated Hospital of Guangxi Medical University; b Department of Chemotherapy, Affiliated Tumor Hospital of Guangxi Medical University, Nanning, China.

**Keywords:** acute pancreatitis, ghrelin, obestatin, predictive value, severity

## Abstract

The roles of ghrelin and obestatin in AP remain controversial.

This study investigates the effects and the predictive value of serum ghrelin and obestatin levels in the early stage of AP.

A total of 193 consecutive patients with AP and 24 healthy controls were included. Patients were divided into mild acute pancreatitis (MAP), moderately severe acute pancreatitis (MSAP), and severe acute pancreatitis (SAP) groups. Serum levels of ghrelin and obestatin were measured on the first, third, and fifth days of hospitalization. The predictive value of serum ghrelin and obestatin levels on the first day in AP was examined using receiver-operating characteristic (ROC) curves.

On the first day of hospitalization, the mean serum ghrelin level was significantly lower in patients with AP than in controls (*P* < .01). The serum ghrelin concentration decreased with increasing AP severity and was lower in patients with SAP than in those with MAP and MSAP (*P* < .05). It increased gradually from the first to the fifth day after treatment. ROC curves demonstrated that the serum ghrelin level on the first day had some predictive value for AP severity (area under the ROC curve = 0.646), with an optimal cut-off value of 87.83 pg/mL. Logistic regression showed that the serum ghrelin level had independent predictive value for non-MAP (odds ratio = 10.94; 95% confidence interval, 5.08–23.55; *P* < .01). The serum obestatin level did not differ significantly between patients with AP and controls and had the limited predictive value for non-MAP (area under the ROC curve = 0.564). However, the serum obestatin concentration showed a “warning” effect regarding AP etiology; on the first day of treatment, it was significantly lower in patients with AP of hypertriglyceridemic etiology than in those with AP of biliary, alcohol-related, and other etiologies (*P* = .05, *P* = .031, and *P* = .029, respectively).

Serum ghrelin and obestatin levels may be related to the progression of AP in the early stage. Only the serum ghrelin level is a potential predictor of AP severity in the early stage. Obestatin may be involved in the pathogenesis of AP caused by hypertriglyceridemia.

## 1 Introduction

Acute pancreatitis (AP) is one of the most common diseases of the digestive system, and its incidence is increasing.^[1]^ The majority of patients with mild acute pancreatitis (MAP) recover within 5 to 7 days with aggressive intravenous hydration and fasting. In about 20% of patients, however, the disease progresses to severe acute pancreatitis (SAP), with up to 10% to 30% mortality.^[2]^ Because of the complex and variable clinical course of the disease, doctors have difficulty with accurate determination of the prognosis of SAP in the early stage, even with the use of advanced scoring systems.^[3,4]^ Early detection of potential SAP is crucial for adequate treatment and the reduction of mortality. For this reason, the prognostic roles of several cytokines and serum biomarkers in AP have been investigated, but the results have been unsatisfactory.^[5,6]^

Ghrelin was first discovered and isolated from rat stomach X/A-like cells.^[7]^ It can stimulate growth hormone secretion by binding to the growth hormone receptor. Initial studies focused on its roles in endocrine, energy balance, appetite, and gastrointestinal regulation.^[8,9]^ Recently, the anti-inflammatory and immunomodulatory activities of ghrelin, including in AP, have been examined.^[10,11]^ Some studies have shown that ghrelin levels are related to the progression of AP, but cut-off values for the effective concentration have been inconsistent.^[12,13]^ Thus, more studies of the effects of serum ghrelin level on AP are needed.

Obestatin, derived from the same prohormone as ghrelin, contributes to the reduction of gastrointestinal motility, appetite suppression, weight loss, and increased memory function in mammals; these roles contrast with the functions of ghrelin.^[14,15]^ The administration of obestatin inhibited the development of AP in a rat model,^[16,17]^ but few studies have investigated the role of endogenous obestatin in human patients with AP.^[18]^ Thus, this study was designed to assess serum ghrelin and obestatin levels in patients with AP, and to determine whether these 2 indexes have predictive value as biomarkers in AP.

## 2 Materials and methods

### 2.1 Participants and baseline data collection

The Ethics Committee of the First Affiliated Hospital of Guangxi Medical University approved this study, and all subjects provided written informed consent. Patients with AP who were admitted to our Department of Gastroenterology between November 2012 and February 2016 were recruited consecutively. AP diagnoses were based on a history of acute abdominal pain, sometimes radiating through to the back, and a 3-fold increase in serum amylase activity or confirmation of pancreatitis by computerized tomography (CT). Patients aged < 18 years; those for whom >48 hours elapsed between the onset of typical abdominal symptoms and study inclusion; those with tumors of the pancreas or other organs; and patients with diabetes mellitus, hypertension, and/or epilepsy were excluded. Healthy volunteers matched with patients according to age, sex, and body mass index (BMI) were included as controls.

The Bedside Index for Severity in Acute Pancreatitis (BISAP) was completed within 24 hours of each patient's admission, and the Ranson criteria were applied within 48 hours of admission.^[19,20]^ All patients underwent contrast-enhanced CT within 48 hours of admission. Modified Computed Tomography Severity Index (MCTSI) scores were calculated within 48 hours after CT examination. Each clinical score was calculated based on the most extreme laboratory data obtained within 48 hours of admission.

### 2.2 Definitions

AP diagnoses were based on the revised Atlanta classification.^[21,22]^ MAP was defined as the absence of organ failure and local or systemic complications. Moderately severe acute pancreatitis (MSAP) was defined as the presence of local and/or systemic complications lasting <48 hours, and/or exacerbation of comorbidity. SAP was defined as persistent organ failure (lasting >48 hours) and 1 or more local complications, according to the Atlanta classification.^[21]^ BMI was calculated as weight (in kilograms) divided by height (in meters) squared. Non-MAP was defined as cases of MSAP and SAP. The etiology of AP was classified as biliary, alcohol related, hypertriglyceridemia, and other (including unknown).

### 2.3 Measurement

Venous blood samples were collected from subjects after overnight fasting. Blood samples were collected from patients on the first, third, and fifth days of hospitalization. The samples were centrifuged immediately, and the serum was stored at **–**80°C until analysis. The serum levels of neutrophils, albumin, calcium, and triglycerides, and the white blood cell (WBC) count, were measured in samples collected on the first day of hospitalization. The serum levels of obestatin and ghrelin were determined using RayBio's Human Enzyme Immunoassay kit (RayBiotech, Norcross).

### 2.4 Statistical analysis

Statistical analysis was performed using SPSS 16.0 software (SPSS Inc., Chicago, IL). Continuous variables are presented as means and standard deviations. Data from patients and controls were compared using Student's *t* test. Differences in ghrelin and obestatin levels among groups were examined using analysis of variance. The sensitivity and specificity of each index were evaluated. A receiver-operating characteristic (ROC) curve was used to describe the performance of continuous variables. The predictive accuracy of ghrelin and obestatin levels in patients with AP was examined using the area under the receiver-operating characteristic curve (AUC). Optimal cut-off values were determined from ROC curve analyses by calculating the minimum distances from graph coordinates with maximum sensitivity and specificity. The AUC results were examined using multivariate logistic regression analyses with forced inclusion of explanatory variables. Multiple comparisons of variance were adjusted for age; WBC count; neutrophil, albumin, calcium, and triglyceride levels; and Ranson, BISAP, and MCTSI scores according to the etiology and severity of AP. To further test the predictive value of non-MAP, we used serum ghrelin and obestatin data from the first day. The significance level was set to *P* < .05.

## 3 Results

### 3.1 Baseline characteristics of subjects

Table 1 shows the baseline characteristics of subjects included in the study. A total of 193 patients (121 males, 72 females; mean age, 49.8 ± 14.7 years) with AP (MAP, n = 94; MSAP, n = 70; SAP, n = 29) were recruited. The time from the first symptom onset to hospital admission ranged from 6 to 48 hours. Twenty-four healthy controls (15 males, 9 females; mean age, 51.8 ± 14.2 years) were included in the study (Table 1).

**Table 1:**
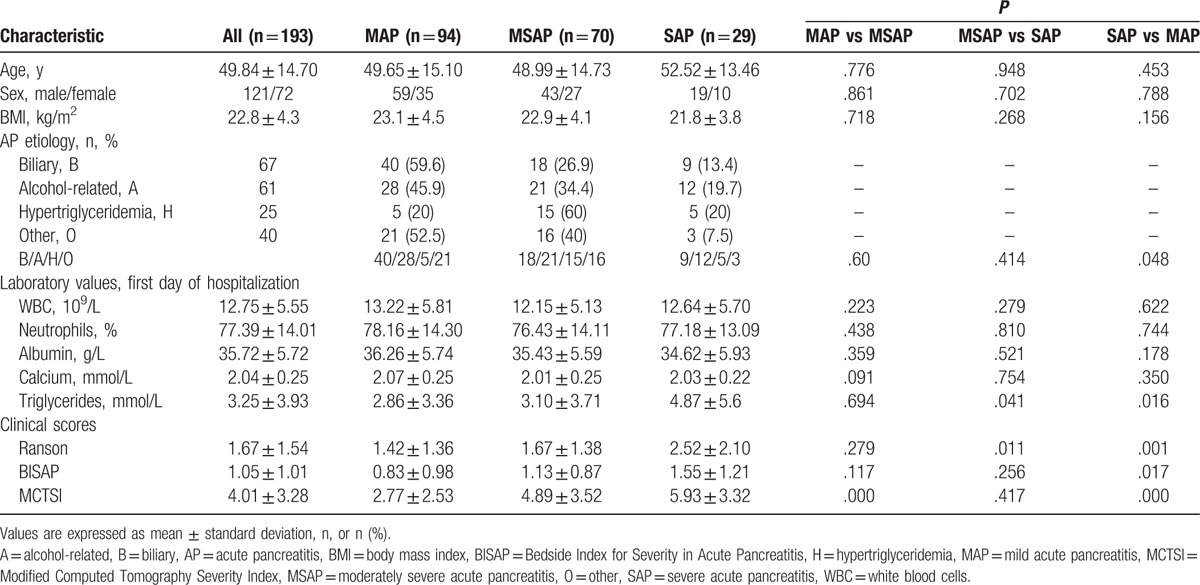
Baseline characteristics of patients according to AP severity.

### 3.2 Serum ghrelin and obestatin levels according to AP severity

**Figure 1:**
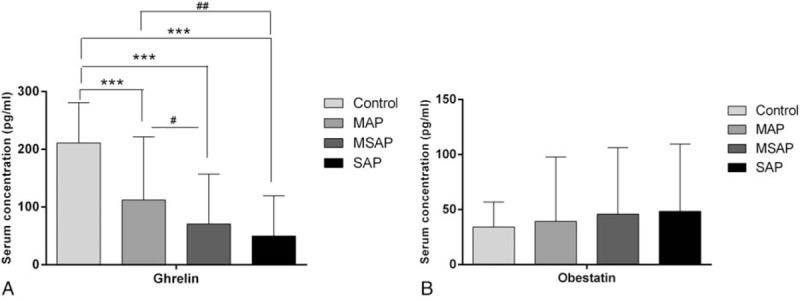
Serum levels of ghrelin (A) and obestatin (B) on the first day of hospitalization in patients with AP. Values are expressed as means ± standard deviations. The significance between the AP group and the control group, ^∗^*P* < .05, ^∗∗^*P* < .01, ^∗∗∗^*P* < .001; the significance between AP groups, ^#^*P* < .05, ^##^*P* < .01, ^###^*P* < .001. AP = acute pancreatitis, MAP = mild acute pancreatitis, MSAP = moderately severe acute pancreatitis.

Serum ghrelin levels were much higher in healthy controls than in patients with MAP, MSAP, and SAP (all *P* < .001). They decreased with increasing AP severity and were significantly higher in patients with MAP than in those with MSAP and SAP (*P* < .05 and *P* < .01, respectively, Fig. 1A, Table 2). No significant difference in the serum ghrelin level was found between patients with MSAP and those with SAP (Fig. 1A, Table 2). After treatment, this concentration increased gradually from the first day to the fifth day. Serum ghrelin levels on the third day were lower in patients with SAP than in those with MAP (*P* < .01); no difference was observed between patients with MSAP and those with SAP. On the fifth day after treatment, the serum ghrelin concentration was lower in patients with SAP than in those with MSAP and MAP (*P* < .05 and *P* < .01, respectively; Table 2). The serum ghrelin concentration thus varied with AP severity.

**Table 2:**
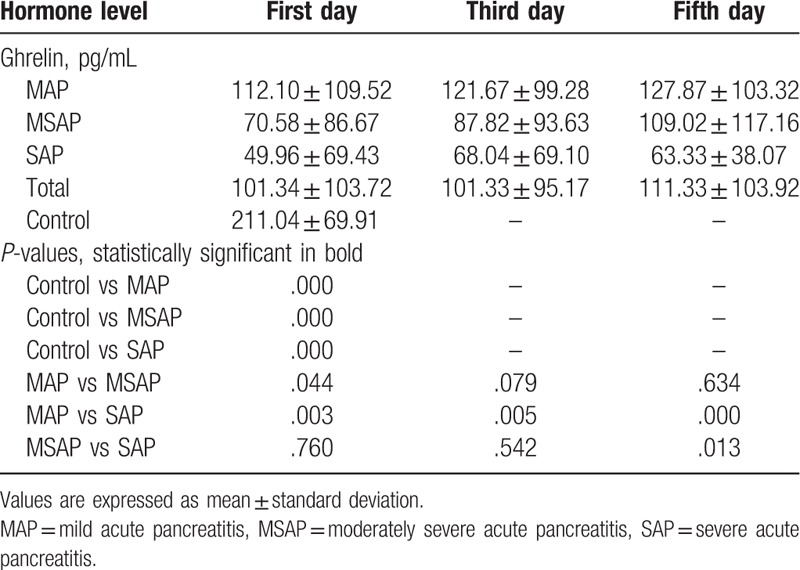
Changes in ghrelin levels of hospitalization.

In contrast to ghrelin, obestatin levels tended to be higher in patients with AP than in controls, but the difference was not significant. Serum obestatin levels did not differ among patients with MAP, MSAP, and SAP (Fig. 1B, Table 3). The obestatin concentration decreased from the first day to the fifth day after treatment, and levels on the third and fifth days did not differ according to AP severity (Table 3). Thus, the serum obestatin level was not correlated with AP severity.

**Table 3:**
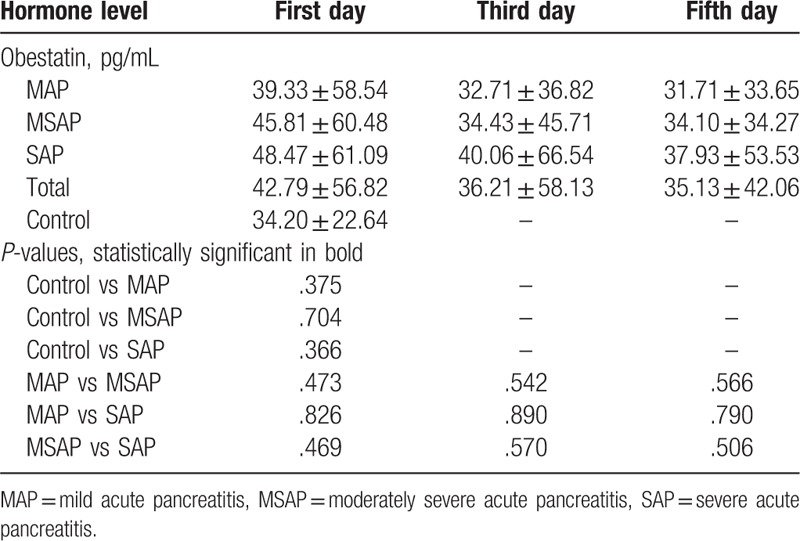
Changes in obestatin levels of hospitalization.

### 3.3 Differences in variables according to AP etiology

**Figure 2:**
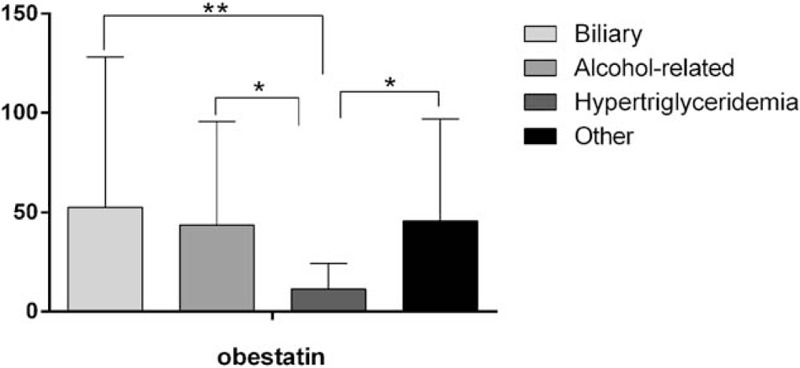
Serum levels of obestatin on the first day of hospitalization according to AP etiology. Values are expressed as means ± standard deviations. ^∗^*P* < .05, ^∗∗^*P* < .01. AP = acute pancreatitis.

Serum obestatin levels on the first day were significantly lower in patients with AP of hypertriglyceridemic etiology than in those with AP of biliary, alcohol-related, and other etiologies (*P* = .005, *P* = .031, and *P* = .029, respectively) (Fig. 2 and Table 4). BISAP scores were significantly higher in the hypertriglyceridemia etiology group than in the other 3 etiology groups (all *P* < .05). Significantly fewer patients with MAP were placed in the hypertriglyceridemia etiology group than in the other 3 etiology groups (*P* < .05) (Table 1). No difference among etiology groups was found in the serum ghrelin level on the first day, age, WBC count, neutrophil, albumin, or calcium level, or Ranson or MCTSI score (Table 4).

**Table 4:**
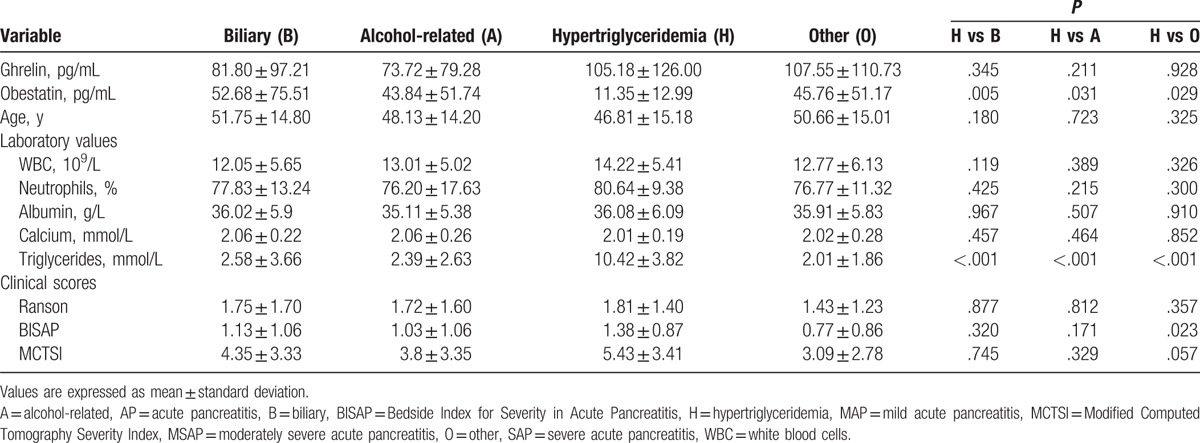
Characteristics on the first day of hospitalization according to AP etiology.

### 3.4 Predictive value of ghrelin and obestatin levels for AP severity

**Figure 3:**
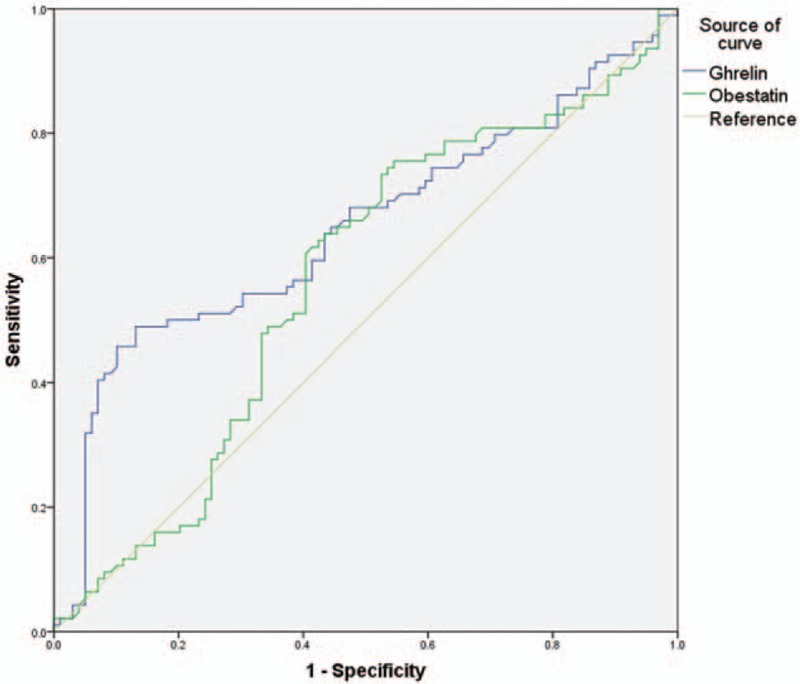
Receiver operating characteristic curves showing the predictive value of serum ghrelin and obestatin levels on the first day of hospitalization for the severity of AP. AP = acute pancreatitis.

Only the ghrelin level showed good predictive value for AP severity (AUC = 0.646); the optimal cut-off value was 87.83 pg/mL, with sensitivity and specificity values of 0.489 and 0.869, respectively (*P* < .01). The obestatin level had little predictive value (AUC = 0.564, *P* = .122; Fig. 3). The results further confirmed that ghrelin was a potentially predictor for the diagnosis and treatment of AP.

### 3.5 Risk factors for non-MAP

In logistic regression models, the serum ghrelin level < 87.83 pg/mL was an independent risk factor for non-MAP (odds ratio = 10.94; 95% confidence interval, 5.08**–**23.55; *P* < .01) (Table 5). The serum obestatin level was not an independent risk factor for non-MAP.

**Table 5:**
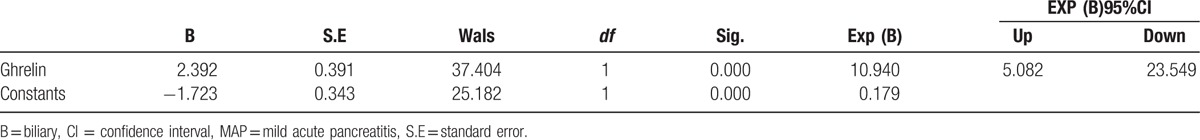
Risk factors for non-MAP.

## 4 Discussion

In the present study, we measured serum ghrelin and obestatin levels in patients with early-stage AP and explored their predictive value for AP severity. Serum ghrelin levels were lower and serum obestatin levels were higher in patients with AP than in healthy controls, suggesting that both hormones are involved in the development of AP. In addition, serum ghrelin and obestatin levels differed according to AP etiology. Using ROC analysis, we determined that a cut-off value of 87.83 pg/mL for the serum ghrelin level had some predictive value for non-MAP. Logistic regression further verified the predictive value of ghrelin for non-MAP, suggesting that ghrelin is a potentially valuable target for the diagnosis and treatment of SAP.

Ghrelin has been reported to regulate central and peripheral lipid metabolism.^[23]^ It was recently shown to play an anti-inflammatory role in some critical illnesses, including AP.^[24]^ Administration of ghrelin had a protective effect on AP of diverse causes, accompanied by decreased serum levels of pro-inflammatory factors, improvement in pancreatic blood flow, and increased DNA synthesis.^[17,25]^ However, reports on serum ghrelin levels in patients with AP have been inconsistent. In our study, serum ghrelin levels at admission were significantly lower in patients with AP than in healthy controls. This result is in agreement with the findings of Panek et al,^[26]^ but contrasts with the results of Daniel et al,^[13]^ who found higher serum ghrelin levels in patients with AP than in controls. This discrepancy may be due to differences in the timing of blood sample collection and AP etiology.

Obestatin is receiving increasing attention because it plays a crucial role in tissue and circulating lipid metabolism. Several studies have shown that acute obestatin treatment and chronic treatment with native or modified obestatin can alter triglyceride levels,^[4,27]^ suggesting that obestatin is involved in the lipid metabolism. In this study, serum obestatin levels in patients with AP were elevated at admission, but were not correlated with AP severity; these findings are consistent with those of Kanat et al.^[18]^ Unlike serum ghrelin levels, serum obestatin levels differed significantly between patients with AP caused by hypertriglyceridemia and those with AP of other etiologies. In addition, these patients had higher BISAP, Ranson, and MCTSI scores than did patients with AP of other etiologies, and the hypertriglyceridemia etiology group included significantly fewer patients with MAP than did the other 3 etiology groups. These findings indicate that patients with AP caused by hypertriglyceridemia tend to develop severe disease.

Several limitations of this study should be noted. First, we did not measure serum ghrelin and obestatin levels at the time of discharge; thus, we do not know whether these levels had returned to normal, which would have provided useful information about patients’ recovery. Second, this study included a large proportion of patients with non-MAP because our hospital is the largest in the region, which could lead to selection bias. Third, although we examined a larger group of patients than included in other published studies, the samples of patients with AP of different etiologies were small, which limited subgroup analyses. Fourth, although we failed to show that the serum obestatin level has predictive value for non-MAP, the inclusion of more patients with AP caused by hypertriglyceridemia might improve the predictive performance of this variable. Thus, our results should be interpreted with caution, and the predictive value of serum ghrelin and obestatin levels in AP should be examined further.

## 5 Conclusions

In summary, this study investigated the role of serum ghrelin and obestatin in early-stage AP. It demonstrated that serum ghrelin and obestatin may be involved in the development of AP. The serum ghrelin level on the first day of hospitalization was related to AP degree and is thus a potential predictor of AP severity. In contrast, the results of the present study do not support the value of obestatin as an indicator of AP severity. However, obestatin levels differed significantly between patients with AP caused by hypertriglyceridemia and those with AP of other etiologies, which suggests that this marker may have a “warning” effect with regard to AP development. The roles of ghrelin and obestatin in AP need to be confirmed with multicenter studies including more patients with AP.
